# Supplementary material for: ABO Blood Groups, RhD Factor and Their Association with Subclinical Atherosclerosis Assessed by Carotid Ultrasonography
Source: J Clin Med. 2024 Feb 27;13(5):1333. doi: 10.3390/jcm13051333 (PMC10931791; doi:10.3390/jcm13051333)
Supplement: Supplementary file 1 [file jcm-13-01333-s001.zip › jcm-2863162-supplementary.pdf]

Table S1: Baseline Characteristics stratified for blood groups and presented separately for men and women.

	O	Non-O	*P*-value[1]	RhD+	RhD-	*P*-value[1]
**Men**						
N	469	778		1056	191	
Age [years]	59.9 (50.0;60.0)	59.9 (50.06;60.03)	0.21	59.9 (50.1;60.0)	59.9 (50.0;60.0)	0.66
BMI [kg/m²]	27.5 (25.3;30.7)	27.5 (25.2;30.6)	0.81	27.4 (25.2;30.5)	28.1 (25.4;31.4)	0.11
Waist [cm]	100.5 (94.0;109.0)	100.0 (94.0;108.0)	0.99	100.0 (94.0;108.0)	101.0 (94.0;110.0)	0.23
SBP [mmHg]	130.0 (120.0;140.0)	130.0 (121.0;140.0)	0.82	130.0 (120.0;140.0)	132.0 (120.0;140.0)	0.79
Hypertension (%)	58.8	58.5	0.97	58.3	60.2	0.68
Diabetes (%)	10.1	8.3	0.31	8.9	9.4	0.92
Smoking (%)	12.0	13.2	0.58	12.6	12.7	1.00
Snus (%)	25.4	28.1	0.34	26.6	29.6	0.45
Using statin (%)	16.1	17.3	0.67	16.5	18.5	0.6
CIMT [mm]	0.75 (0.65;0.88)	0.74 (0.65;0.87)	0.92	0.74 (0.65;0.87)	0.75 (0.65;0.89)	0.88
Plaque absence (%)	49.9	46.1	0.22	47.0	50.8	0.37
Plaque unilateral (%)	26.7	25.8	0.79	26.8	22.5	0.2
Plaque bilateral (%)	23.5	28.0	0.09	26.2	26.7	0.8
**Women**						
N	633	1049		1445	237	
Age [years	59.9 (50.0;60.0)	59.9 (50.0;60.0)	0.84	59.9 (50.0;60.0)	59.9 (50.1;60.0)	0.52
BMI [kg/m²]	26.5 (23.6;30.4)	26.7 (23.7;30.5)	0.33	26.6 (23.7;30.5)	26.7 (23.1;29.8)	0.29
Waist [cm]	91.0 (82.0;100.0)	92.0 (83.0;101.0	0.37	91.0 (83.0;101.0)	92.0 (83.0;100.0	0.65
SBP [mmHg]	125.0 (115.0;135.0)	127.0 (115.0;138.0)	0.07	126.0 (115.0;136.0)	124.0 (115.0;136.0)	0.42
Hypertension (%)	44.2	47.7	0.18	45.49	51.48	0.10
Diabetes (%)	4.9	6.6	0.18	5.8	6.9	0.60
Smoking (%)	12.3	13.3	0.62	12.2	17.3	**0.04***
Snus (%)	9.8	10.5	0.74	9.9	12.1	0.38
Using statin (%)	8.7	8.8	1.00	8.9	7.6	0.58
CIMT [mm]	0.68 (0.62; 0.78)	0.68 (0.61; 0.78)	0.81	0.68 (0.61; 0.78)	0.67 (0.61; 0.77)	0.25
Plaque absence (%)	60.0	63.6	0.16	61.9	64.6	0.47
Plaque unilateral (%)	24.2	21.8	0.22	22.9	21.5	0.60
Plaque bilateral (%)	15.6	14.6	0.43	15.2	13.9	0.60

Continuous variables presented as medians (25th to 75th percentiles) and categorical variables as percent

1. Calculated with Mann-Whitney U test for continuous variables and Chi²-test for categorical. *P*-value < 0.05 in bold.

Table S2: Drop-out analysis comparing excluded and included participants.

	Included	Excluded	*P*-value[1]
N	2929	603	
Male N (%)	1247 (42.6)	415 (68.8)	**<0.001**
Age [years]	59.9 (50.0;60.0)	59.9 (50.0;60.03)	0.92
BMI [kg/m^2]	27.1 (24.3;30.5)	26.84 (24.3;30.1)	0.36
Waist [cm]	96.0 (87.0;105.0)	97.0 (88.8;105.0)	0.17
SBP [mmHg]	128.0 (118.0;139.0)	130.00 (120.0;140.0)	**<0.001**
Hypertension (%)	51.5	53.1	0.53
Diabetes (%)	7.1	5.8	0.27
Smoking (%)	12.8	11.8	0.53
Snus (%)	17.1	21.9	**0.006**
Using statin (%)	11.7	7.6	**0.004**
CIMT [mm]	0.71 (0.63;0.82)	0.72 (0.63;0.82)	0.30
Plaque abscence (%)	56.0	51.9	0.07
Plaque on one side (%)	24.2	24.0	0.57
Plaque on two sides (%)	19.8	24.0	**0.02**

Continuous variables presented as medians (25th to 75th percentiles) and non-continuous variables as percent

1. Calculated with Mann-Whitney U test for continuous variables and Chi^2-test for categorical. *P*-value < 0.05 in bold.

Table S3 the impact of RhD on CIMT [B (CI95%)] assessed in non-O and O blood group separately

**CIMT**	**O**		**Non-O**	
	RhD+	RhD-	RhD+	RhD-
All age groups (N)	948	154	1553	274
B (CI 95%)	ref	0.99 (0.95; 1.02)	ref	1.00 (0.97; 1.02)
B (CI 95%)[1]	ref	0.99 (0.96; 1.02)	ref	0.92 (0.97;1.01)
40-year-old (N)	80	13	105	21
B (CI 95%)	ref	1.08 (1.00; 1.17))	ref	**1.09 (1.01; 1.17)**
B (CI 95%)[2]	ref	**1.04 (1.01; 1.17)**	ref	**1.08 (1.01; 1.15)**
50-year-old (N)	256	46	451	69
B (CI 95%)	ref	0.96 (0.67;1.43)	ref	1.00 (0.95; 1.04)
B (CI 95%)[2]	ref	0.96 (0.91; 1.01)	ref	1.00 (0.96; 1.04)
60-year-old (N)	612	95	997	184
B (CI 95%)	ref	0.99 (0.95; 1.04)	ref	0.98 (0.95; 1.01)
B (CI 95%)[2]	ref	0.99 (0.95; 1.03)	ref	0.98 (0.95; 1.01)

1. Adjusted for sex and age . 2. Adjusted for sex.

B = unstandardized B. B is interpreted as the ratio of geometric mean of CIMT compared to the reference.

Thus, B > 1 implies that mean CIMT was higher among RhD- compared to RhD+

Table S4. Comparison of ABO blood group (Non-O vs O) and RhD (RhD- vs RhD+) and their association with CIMT [B (CI95%)]. In 50- and 60-year-old participants that reported heredity for CVD when aged 40 years.

CIMT	O	Non-O	RhD+	RhD−
50-year-old (N)	63	90	133	20
B (CI 95%)	ref	0.99 (0.94; 1.05)	ref	0.97 (0.90; 1.06)
B (CI 95%)[1]	ref	1.00 (0.94; 1.05)	ref	0.97 (0.89;1.05)
60-year-old (N)	89	163	220	32
B (CI 95%)	ref	1.01 (0.96; 1.06)	ref	1.01 (0.94; 1.09)
B (CI 95%)[1]	ref	1.01 (0.97; 1.07)	ref	1.01 (0.94; 1.08)

1. Adjusted for sex

B = Unstandardized B. B is interpreted as the ratio of geometric mean of CIMT compared to the reference. Thus, B > 1 implies that mean CIMT was higher among Non-O or RhD- compared to O or RhD+, respectively.
